# Regionally diverse astrocyte subtypes and their heterogeneous response to EAE

**DOI:** 10.1002/glia.23954

**Published:** 2020-12-17

**Authors:** Malte Borggrewe, Corien Grit, Ilia D. Vainchtein, Nieske Brouwer, Evelyn M. Wesseling, Jon D. Laman, Bart J. L. Eggen, Susanne M. Kooistra, Erik W. G. M. Boddeke

**Affiliations:** ^1^ Department of Biomedical Sciences of Cells & Systems Section Molecular Neurobiology, University Medical Center Groningen, University of Groningen Groningen The Netherlands; ^2^ Department of Psychiatry and Weill Institute for Neurosciences University of California San Francisco California USA; ^3^ Center for Healthy Ageing, Department of Cellular and Molecular Medicine University of Copenhagen Copenhagen Denmark

**Keywords:** astrocyte subpopulations, astrogliosis, cellular heterogeneity, homeostasis, RNA sequencing

## Abstract

Astrocytes fulfil many functions in the central nervous system (CNS), including contribution to the blood brain barrier, synapse formation, and trophic support. In addition, they can mount an inflammatory response and are heterogeneous in morphology and function. To extensively characterize astrocyte subtypes, we FACS‐isolated and gene expression profiled distinct astrocyte subtypes from three central nervous system regions; forebrain, hindbrain and spinal cord. Astrocyte subpopulations were separated based on GLAST/SLC1A3 and ACSA‐2/ATP1B2 cell surface expression. The local brain environment proved key in establishing different transcriptional programs in astrocyte subtypes. Transcriptional differences between subtypes were also apparent in experimental autoimmune encephalomyelitis (EAE) mice, where these astrocyte subtypes showed distinct responses. While gene expression signatures associated with blood–brain barrier maintenance were lost, signatures involved in neuroinflammation and neurotoxicity were increased in spinal cord astrocytes, especially during acute disease stages. In chronic stages of EAE, this reactive astrocyte signature was slightly decreased, while obtaining a more proliferative profile, which might be relevant for glia scar formation and tissue regeneration. Morphological heterogeneity of astrocytes previously indicated the presence of astrocyte subtypes, and here we show diversity based on transcriptome variation associated with brain regions and differential responsiveness to a neuroinflammatory insult (EAE).

## Main Points

- ACSA and GLAST can be used to FACS‐sort astrocyte subtypes
- Astrocytes in forebrain and hindbrain consist of two subtypes based on GLAST expression
- Spinal cord astrocytes are reactive in acute EAE and are more proliferative in chronic stages

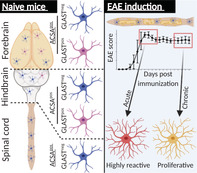

## 1 INTRODUCTION

Astrocytes fulfil numerous essential functions in the central nervous system (CNS), including structural and metabolic support that shape myelination, blood brain barrier (BBB) formation, and synaptic transmission (Pekny et al., 2016; Xin & Bonci, 2018). Consistent with this wide range of features, astrocytes exhibit considerable functional and molecular heterogeneity (Boisvert, Erikson, Shokhirev, & Allen, 2018; Matyash & Kettenmann, 2010; Xin & Bonci, 2018; Zhang & Barres, 2010). Regional differences include distinct expression and activity of potassium channels, transporters and gap junctions (Lee, Kim, Cornell‐Bell, & Sontheimer, 1994; Xin & Bonci, 2018), morphology (Chai et al., 2017), and cellular functions such as proliferative capacity (Emsley & Macklis, 2006). Furthermore, the astrocyte marker GFAP displays region‐dependent differences in expression (Zhang & Barres, 2010), and gene expression patterns in astrocytes follow the dorsoventral axis (Morel et al., 2017). Hence, astrocytes feature molecular and functional heterogeneity that is shaped by local environmental cues of different anatomical regions.

In addition to interregional differences, astrocytes also display intraregional heterogeneity (Farmer & Murai, 2017; John Lin et al., 2017; Morel et al., 2019; Zeisel et al., 2015). Differential expression of GLT‐1/SLC1A2 defines astrocyte subtypes which are transcriptionally distinct (Morel et al., 2019). In addition, astrocyte subtypes found across anatomical regions exhibit functional differences in synaptogenesis support (John Lin et al., 2017). These subtypes are also associated with glioma disease symptoms, suggesting differential contribution of astrocyte subtypes to CNS disease (John Lin et al., 2017).

During disease and aging, homeostatic astrocyte functions can get impaired, thereby contributing to CNS dysfunction (Pekny et al., 2016). Conversely, reactive astrocytes can also provide protective signals to contain local damage and to support regeneration (Alilain, Horn, Hu, Dick, & Silver, 2011; Liddelow & Barres, 2017). Following lipopolysaccharide (LPS) or middle cerebral artery occlusion (MCAO), mouse astrocytes adopt two transcriptionally distinct reactive phenotypes (Zamanian et al., 2012). LPS induces genes associated with neurotoxic effects (known as A1 astrocytes), whereas astrocytes after MCAO predominantly express protective and to a lesser extent neurotoxic genes (known as A2 astrocytes) (Rakers et al., 2019; Zamanian et al., 2012). The LPS‐reactive astrocyte phenotype is induced by activated microglia and markers of this phenotype are expressed as a result of aging and in Alzheimer's‐, Huntington's‐, Parkinson's‐disease, and multiple sclerosis (MS) (Boisvert et al., 2018; Clarke et al., 2018; Liddelow et al., 2017; Orre et al., 2014). A1 astrocytes share similarities with an astrocyte subpopulation that expands in Alzheimer's disease, called disease‐associated astrocytes (Habib et al., 2020).

In experimental autoimmune encephalomyelitis (EAE), a widely used mouse model for MS, astrocytes are implicated in disease development, inflammatory response, immune cell recruitment, and remyelination (Brambilla et al., 2014; Itoh et al., 2017; Rothhammer et al., 2018; Wang et al., 2013). Astrocytes undergo reactive gliosis and upregulate immune‐related genes (Wheeler et al., 2020), whereas the expression of cholesterol synthesis genes is decreased (Itoh et al., 2017). Spinal cord astrocytes are affected most by EAE compared to other anatomical regions, highlighting interregional heterogeneity also in diseased states (Itoh et al., 2017). A detailed genome‐wide characterization of transcriptional changes in astrocytes is lacking and the role of astrocyte subtypes in EAE is presently unknown.

Here, we assessed gene expression profiles of astrocyte subtypes defined by anatomical regions and surface expression of astrocyte markers GLAST/SLC1A3 and ACSA‐2/ATP1B2. We delineated differential contribution of these astrocyte subtypes in EAE and generated a transcriptional blueprint of spinal cord and hindbrain astrocytes during the progression of disease.

## 2 MATERIALS AND METHODS

### 2.1 Animals

All animal experiments were approved by the Netherlands Central Committee for Animal Experiments and the University of Groningen. For experiments related to astrocyte heterogeneity, FVB/N wildtype were used, whereas C57BL/6 mice were used for EAE experiments. Mice were housed SPF in groups in makrolon cages with ad libitum access to water and food, and a 12 h light–dark cycle (8 p.m. lights off, 8 a.m. lights on).

### 2.2 EAE induction and scoring

For induction of EAE, 10‐week old female C57BL/6 mice (Harlan, The Netherlands) were immunized with MOG35‐55 in complete Freund's adjuvant (CFA) (Hooke, EK‐2110). Mice were injected with pertussis toxin on the day of immunization and 24 h later. Animals were monitored daily for development of EAE and sacrificed at score 1 (limp tail), score 4 (complete hind leg paralysis) and chronic disease.

### 2.3 Astrocyte isolation

Mice were perfused with 0.9% saline under isoflurane anesthesia. Brains and spinal cords were isolated and collected in HBSS (Gibco, 14,170) supplemented with 15 mM HEPES (Lonza, BE17‐737E) and 0.6% glucose (Sigma‐Aldrich, G8769) (= Medium A). Tissues were sliced and incubated at 37°C for 60 min in medium A containing 0.25% Trypsin/EDTA (Lonza, BE02‐007E) and 0.5 mg/mL DNase (Roche, 10,104,159,001). Enzymatic reactions were neutralized by addition of 10% fetal bovine serum (FBS) (Bovogen Biologicals, SFBS). Subsequently, the suspension was gently triturated, followed by filtration over a 100 μm strainer (Falcon, 352,360) to obtain a single cell suspension. Cells were centrifuged at 300*g* for 10 min at 4°C. After removal of the supernatant, the pellet was resuspended in 24.5% percoll (GE Healthcare, 17–0891‐01), 40 mM NaCl and 77% myelin gradient buffer (5.6 mM NaH2‐PO4·H2O, 20 mM Na2HPO4·2H2O, 140 mM NaCl, 5.4 mM KCl, 11 mM glucose, pH 7.4). A layer of PBS (Lonza, BE17‐512F) was added on top, after which the gradient was centrifuged at 800*g* for 20 min at 4°C with breaks off. The supernatant was removed, and the pellet resuspended in medium A without phenol red supplemented with 1 mM EDTA (Invitrogen, 15,575–038). After Fc receptors were blocked using anti‐mouse CD16/CD32 (1:100, eBioscience, 14–0160‐82) for 10 min, cells were incubated for 30 min on ice with GLAST‐APC (1:10, Miltenyi, 130–095‐814), ACSA‐2‐PE (1:10, Miltenyi, 130–102–365), CD11B‐PE‐Cy7 (1:150, eBioscience, 25–0112‐81), and CD45‐FITC (1:200, eBioscience, 11–0451‐85) antibodies. Next, cells were washed and collected in round bottom tubes, after passing over a 35 μm strainer (Falcon, 352,235). Cells were sorted using a Beckman Coulter MoFlo Astrios cell sorter or a Beckman Coulter MoFlo XDP cell sorter. For subtype experiments, DAPI (Biolegend, 422,801) was added to select for viable cells. For EAE experiments, DAPI and DRAQ5 (Thermo Scientific, 62,251) were added to select for viable cells. Cells were sorted in RNAlater (Qiagen, 76,104), centrifuged 5,000 g for 10 min, and lysed in RLT+ lysis buffer (Qiagen, 74,034). Flow cytometry data was analyzed using Kaluza Analysis (v1.5).

### 2.4 Primary neonatal astrocyte culture

Primary neonatal microglia cultures were prepared as described previously (Schaafsma et al., 2015), which were used to obtain astrocyte cultures. Briefly, cerebrum from postnatal day 0–2 C57BL/6 mice was minced and incubated in trypsin‐containing medium. After trituration and centrifugation of tissue, cells were plated in flasks and medium (DMEM (Gibco, 11,500,416), supplemented with 1 mM sodium pyruvate (Lonza, BE13‐115E), 1x GlutaMAX (Gibco, 35,050,038), 1% Pen/strep (Sigma, P4333) and 10% FBS) was replaced 24 h later. Medium was replaced on day 4, and on day 7 medium supplemented with 33% L929 cell‐conditioned medium (LCCM) was added. Three days after LCCM addition, microglia were harvested through mitotic shake. Astrocytes remained in the flask and were used for experiments.

### 2.5 RNA isolation and RNA sequencing

RNA was extracted from acutely FACS‐isolated astrocytes using the RNeasy Plus Micro kit (Qiagen, 74034) according to the manufacturer's protocol. Astrocytes from up to two mice were pooled for RNA isolation. RNA was extracted from cultured astrocytes with TRIzol (Invitrogen, 15596018) according to manufacturer's instructions and resuspended in water. Total RNA concentration and quality were measured using the Experion Automated Electrophoresis System (Bio‐Rad) in combination with the RNA HighSens Analysis Kit (Bio‐Rad, 7007105) according to manufacturer's protocol. Nondegraded RNA‐samples (RNA integrity number > 5) were selected for subsequent sequencing analysis. Sequencing libraries were manually generated using the QuantSeq 3' mRNA‐Seq Library Prep Kit FWD for Illumina (Lexogen, 015.96). The obtained cDNA fragment libraries were pooled at equal molarities and sequenced on an Illumina HiSeq2500 using default parameters (single read 1x50bp) in pools of multiple samples.

### 2.6 RNA sequencing analysis

#### 2.6.1 Alignment

After trimming of bad quality bases, FASTQ files were aligned to build Mus_musculus.GRCm38.82 reference genome using HISAT (hisat/0.1.5‐beta‐goolf‐1.7.20) with default settings (Kim, Langmead, & Salzberg, 2015). Aligned reads were sorted using SAMtools (SAMtools/1.‐goolf‐1.7.20) (Li et al., 2009) and gene level quantification was done by HTSeq‐count (HTSeq/0.6.1p1) using –mode = union (Anders, Pyl, & Huber, 2015). Quality control metrics for raw sequencing data were calculated using FastQC (FastQC/0.11.3‐Java‐1.7.0_80), and for aligned reads using Picard‐tools (Picard/1.130‐Java‐1.7.0_80).

#### 2.6.2 Differential gene expression analysis

Genes with low expression (total counts<10) were filtered. DEseq2 R‐package (v1.22.2) (Love, Huber, & Anders, 2014) was used for normalization, transformation, and differential gene expression analysis. For PCA plots, counts were transformed using variance stabilizing transformation (VST). Genes were regarded differentially expressed with log2FoldChange > 1 or < −1 and *p*_adj_ < .05. *p*‐values were adjusted using the Benjamini‐Hochberg correction. Overlapping DEGs were visualized in UpSet diagrams (v1.3.3) (Lex, Gehlenborg, Strobelt, Vuillemot, & Pfister, 2014).

#### 2.6.3 Weighted gene co‐expression network analysis

VST‐transformed normalized counts after filtering of low expressed genes (total counts<10) from spinal cord astrocytes of unimmunized mice and during EAE were used for WGCNA. The WGCNA R‐package (v1.68) (Langfelder & Horvath, 2008) was used for the analysis. Genes with missing values and zero variance were filtered prior to network construction (goodSamplesGenes). A signed network was constructed using dissimilarities of topological overlap matrix (1‐TOMsimilarityFromExpr) with a soft threshold power of 6. Modules were computed with a minimum size of 30 and a merge threshold of 0.25, which resulted in 28 modules. Module eigengenes were correlated with EAE stages from unimmunized to chronic EAE and correlation was regarded significant with a *p*‐value<.05.

#### 2.6.4 Gene ontology analysis

Biological process gene ontology (GO) enrichment analysis for DEGs and WGCNA module genes was done using the clusterProfiler R‐package (enrichGO) (v3.10.1) (Yu, Wang, Han, & He, 2012). GO terms were regarded enriched for a list of genes with *q*‐value < .05. *p*‐values were adjusted using the Benjamini–Hochberg correction.

### 2.7 Immunohistochemistry

Tissue was fixed in 4% paraformaldehyde (PFA) for 24 h and cryopreserved in 30% sucrose prior to freezing at −50°C. Sodium citrate (pH 6.0) was used for heat‐induced antigen retrieval in a microwave using a pressure‐cooker. Tissue sections were blocked 1 h in 5% normal serum. GFAP (1:200, Invitrogen, 14‐9892‐82), MHC‐II (1:100, Invitrogen, 14‐5321‐82), and KI67 (1:100, Abcam, ab15580) primary antibodies were diluted in PBS containing 0.1% Triton X‐100 and 1% normal serum and applied at 4°C overnight. Secondary fluorescent antibodies were applied for 1 h at room temperature (RT). Tissue sections were incubated in Bisbenzimide h 33,258 (1:1000, Sigma‐Aldrich, 14530‐100MG) for 15 min. Images were obtained using a Leica SP8 confocal microscope.

## 3 RESULTS

### 3.1 FACS‐isolation of astrocyte subpopulations from distinct regions reveals transcriptional heterogeneity

**FIGURE 1:**
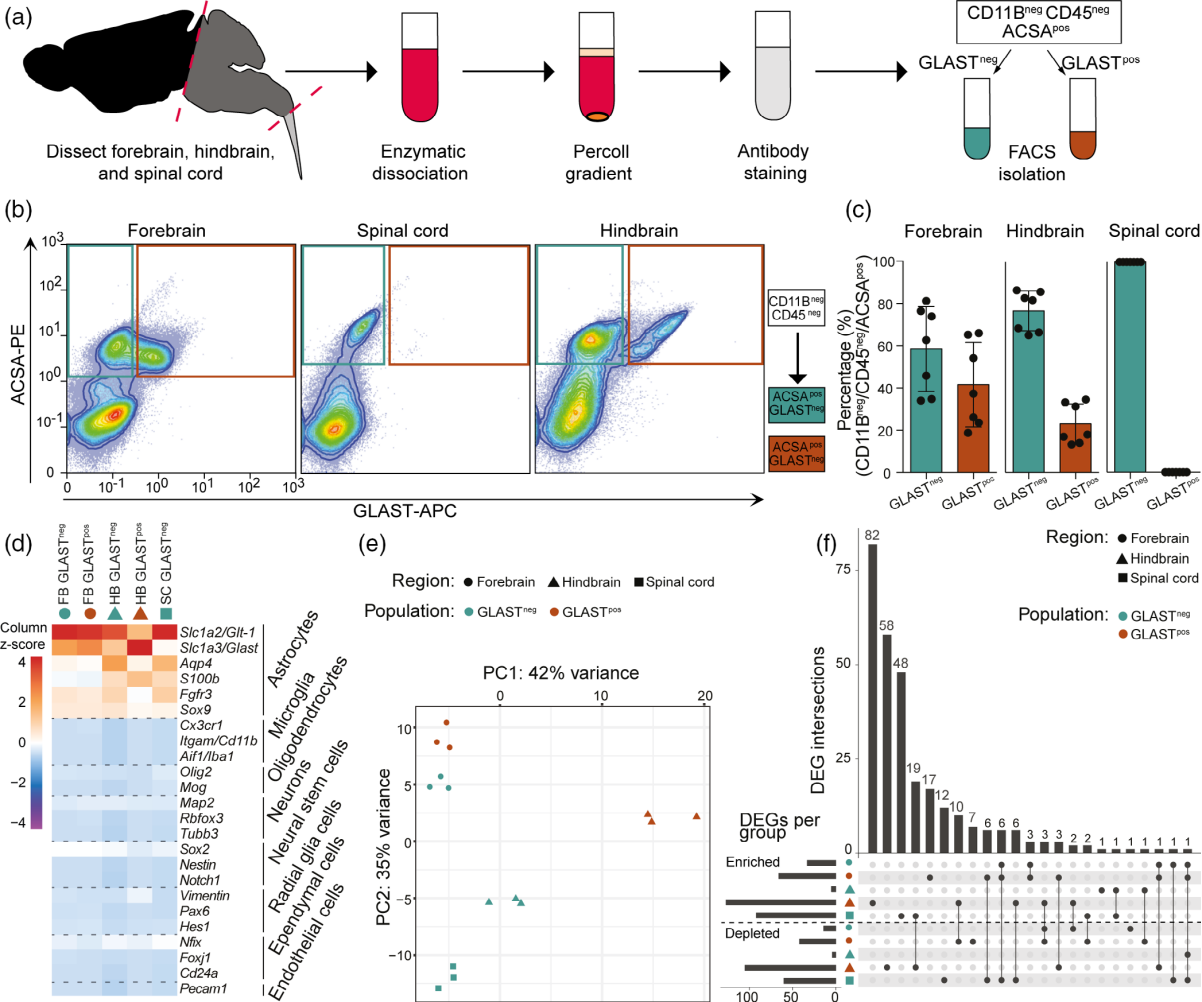
GLAST surface expression and anatomical regions distinguish astrocyte subtypes. (a) Schematic overview of the FACS‐based astrocyte isolation procedure. Astrocytes were isolated from forebrain, hindbrain, and spinal cord. Astrocyte subtypes were selected as CD11B^neg^CD45^neg^ACSA^pos^ events and based on GLAST expression. (b) Representative FACS dot plots of ACSA and GLAST expression in each CNS region (left panel). For complete gating strategies see Figure S1A. Frequency of astrocyte subtypes in different anatomical regions (right panel) as percentages (*n* = 7). Bars indicate mean ± *SD*. (c) Mean expression of the different CNS cell type markers in astrocyte subtypes depicted as column z‐scores. (d) Principal component analysis of astrocyte subtypes in forebrain, hindbrain, and spinal cord. (e) Upset diagram depicting the number and overlap of DEGs comparing all astrocyte subtype to all other subtypes. Bars show the number of enriched and depleted genes (bottom‐left). Overlapping DEGs are illustrated by interconnected dots between groups (bottom‐right), and the number of DEGs are plotted in the bar graph (top)

Methods to isolate astrocytes by FACS without the use of fluorescently tagged transgenes are limited. We developed an antibody‐based approach to isolate pure and intact astrocytes from different brain regions. The mouse CNS was dissected in forebrain (including olfactory bulbs), hindbrain (cerebellum and brain stem), and spinal cord (cervical and thoracic parts) (Figure 1a). CNS cells were labelled using antibodies targeting CD11B, CD45, GLAST/SLC1A3 and ACSA‐2/ATP1B2 (Batiuk et al., 2017; Kantzer et al., 2017) (referred to as ACSA) (Figure 1a). GLAST and ACSA are expressed by astrocytes (Batiuk et al., 2017; Kantzer et al., 2017; Schreiner et al., 2014). After selecting DAPI^neg^ live cells, myeloid cells were excluded based on CD11B and CD45 expression (Figure S1A). In both forebrain and hindbrain, ACSA^pos^ astrocytes were fractionated based on expression of GLAST (GLAST^pos^ and GLAST^neg^); in spinal cord, only GLAST^neg^ (ACSA^pos^) astrocytes were isolated (Figure 1b). Of note, we observed GLAST^pos^ astrocytes in spinal cord (less than 1% of ACSA^pos^ astrocytes), but the numbers were insufficient to perform downstream analysis. *Acsa* is abundantly expressed in all brain regions and the spinal cord as evident from in situ hybridization and spatial transcriptomics, whereas *Glast* expression is regionally diverse in brain, and very low in spinal cord (Figure S1B,C) (10x Genomics, 2019; Allen Institute, 2004, 2008; Lein et al., 2007; Maniatis et al., 2019). All astrocyte populations abundantly expressed established astrocyte markers *Glt‐1/Slc1a2*, *S100b*, *Fgfr3*, *Sox9*, and *Aqp4* (Figure 1(c)). Expression of markers for microglia, oligodendrocytes, neurons, neural stem cells, radial glia cells, ependymal cells, and endothelial cells was low or not detected, indicating that the obtained astrocyte populations were not contaminated by other CNS cell types. These results demonstrate that pure, distinct astrocyte populations were isolated from nontransgenic mice.

Several studies suggest heterogeneity among astrocytes within and between different regions exists (Chai et al., 2017; John Lin et al., 2017; Morel et al., 2019); hence, we performed RNA‐seq on all astrocyte populations from different anatomical regions. Principal component analysis (PCA) indicated clear segregation of astrocytes based on their anatomical origin (forebrain, hindbrain, spinal cord) (Figure 1(d)). We determined genes that were specifically enriched in astrocytes from distinct anatomical regions, compared to all other regions (Figure S2A). Genes that exhibit highest enrichment in forebrain astrocytes (logFC>6, *p*_adj_<.001) were *Dmrta2*, *Chrdl1*, *Prss5*, and *Crym*, while in hindbrain *Mybpc1* and *Wif1* were most enriched (Figure S2A). In spinal cord astrocytes, several homeobox (*Hox*) genes were highly enriched including *Hoxc6*, *Hoxc8‐9*, *Hoxa7*, and *Hoxa9* (Figure S2A). In situ hybridization and spatial transcriptomics data verified that *Hoxa7*, *Hoxa9*, and *Hoxc9* are predominantly expressed in spinal cord, but absent in brain (Figure S2B,C) (10x Genomics, 2019; Allen Institute, 2004, 2008; Lein et al., 2007; Maniatis et al., 2019). Most differentially expressed genes (DEGs) between anatomical regions were detected in spinal cord astrocytes compared to hindbrain and forebrain astrocytes (Figure S2D), suggesting they are transcriptionally most distinct from other regions. Furthermore, we compared the gene expression of astrocytes from different regions to primary neonatal astrocytes after 14 days of in vitro culture, which exhibited extensive differences in their transcriptional profile (Figure S3A,B). Genes involved in “wound healing” and “actin filament organization” were enriched in cultured astrocytes, while genes associated with “synapse organization” and “axon development” were depleted (Figure S3C).

We next investigated potential intraregional differences in astrocyte subtypes and observed segregation of GLAST^pos^ and GLAST^neg^ astrocytes in forebrain and hindbrain, which was more pronounced in hindbrain. Comparison of astrocyte populations revealed distinct transcriptomes with a number of enriched and depleted genes per population (Figure 1(e)). Myelination‐associated GO terms were annotated for genes enriched in spinal cord and genes depleted in hindbrain GLAST^pos^ (Figure S2E). Genes enriched in both forebrain populations were associated with “forebrain development”, and genes enriched in hindbrain GLAST^pos^ astrocytes were annotated with “extracellular matrix (ECM) organization” and “cell‐substrate adhesion” (Figure S2E).

Together these data support pronounced interregional and intraregional heterogeneity in the transcriptomes of astrocytes and suggest that GLAST expression distinguishes distinct astrocyte subtypes.

### 3.2 GLAST^pos^ and GLAST^neg^ astrocytes are transcriptionally distinct

To delineate the differences between GLAST^pos^ and GLAST^neg^ astrocytes, we further assessed their transcriptional profiles and compared their gene signatures with published astrocyte mRNA profiles.

**FIGURE 2:**
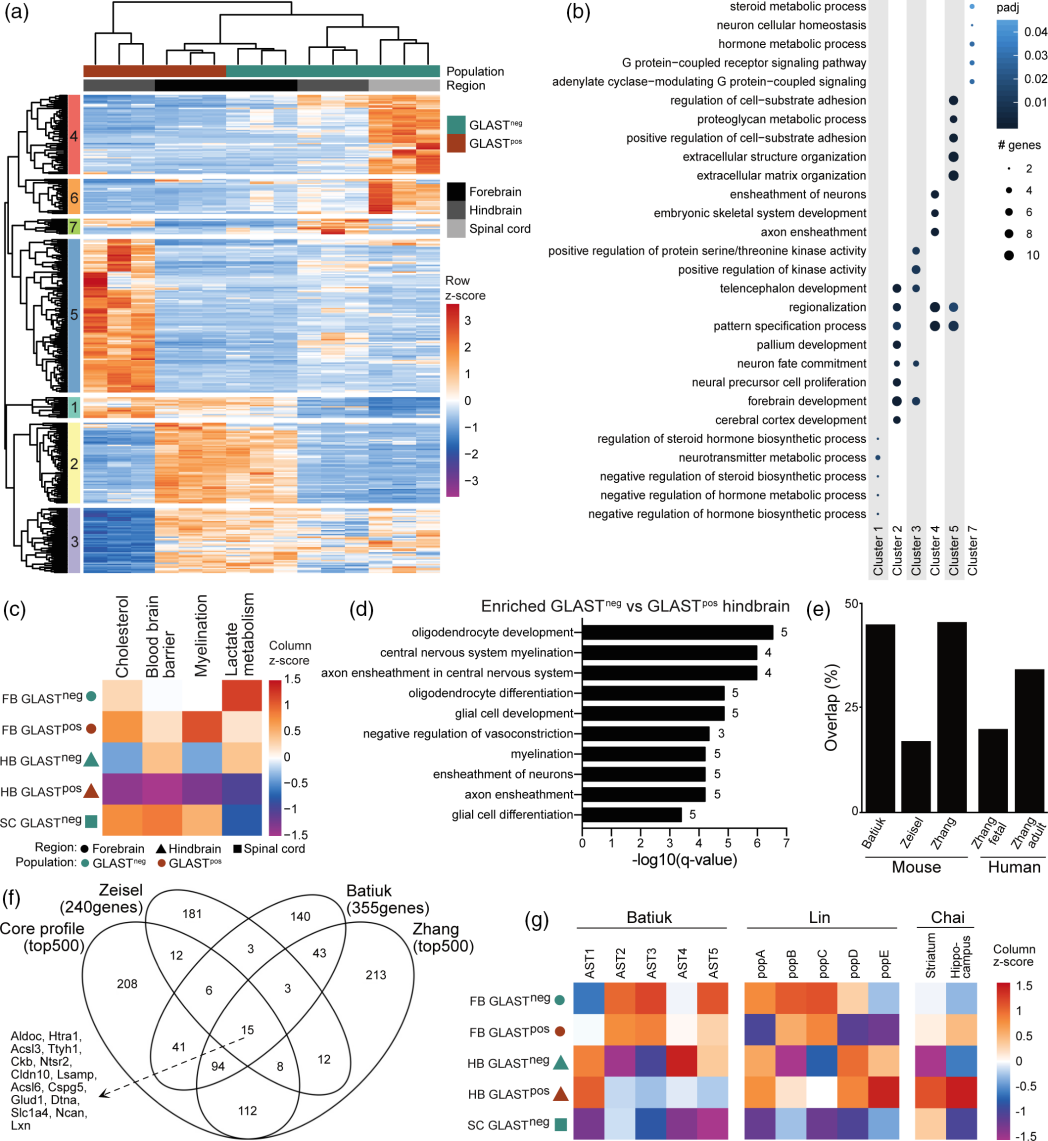
Astrocyte subtypes are transcriptionally distinct. (a) Unsupervised clustering of all genes differentially expressed in each subtype per region compared to all other groups, illustrated as row z‐scores of normalized counts. (b) GO terms enriched in gene clusters from (c). Top five enriched GO terms per cluster are plotted against enrichment of these GO terms in all clusters. Only clusters with significantly enriched GO terms are shown. (c) Mean expression of genes involved in lactate metabolism, myelination, blood brain barrier, and cholesterol synthesis illustrated as z‐scores per group (*n* = 3). For lists of genes see Table S1. (d) GO terms associated with genes enriched in hindbrain (HB) GLAST^neg^ compared to HB GLAST^pos^. Numbers behind bars indicate number of genes per GO category. (e) Percentage overlap of top 500 astrocyte core genes with published astrocyte gene sets (Batiuk et al., 2020; Zeisel et al., 2015; Zhang et al., 2014, 2016). (f) Overlap of top 500 astrocyte core genes with published mouse astrocyte gene sets (Batiuk et al., 2020; Zeisel et al., 2015; Zhang et al., 2014) visualized in a Venn diagram. Genes overlapping in all four datasets are indicated. (g) Mean expression of genes associated with astrocyte subtypes of published datasets (Batiuk et al., 2020; Chai et al., 2017; John Lin et al., 2017) illustrated as z‐score per group (*n* = 3)

Hierarchical clustering was performed on all DEGs (genes enriched and depleted in astrocyte subtypes; Figure 1(e)), which resulted in seven gene clusters of genes based on their expression in astrocyte populations (Figure 2(a)). Clusters 4 and 6 contained genes that were highly expressed in spinal cord and moderately in hindbrain GLAST^neg^ astrocytes (Figure 2(a)). Genes in cluster 4 were associated with “axon ensheathment”, based on GO analysis (Figure 2(b)). Genes in clusters 5 and 1 were enriched for “ECM organization” and “hormone metabolism” and were predominantly expressed in hindbrain GLAST^pos^ astrocytes (Figure 2(a),(b)). GLAST^pos^ and GLAST^neg^ astrocytes in forebrain exhibited similar expression of DEGs that related to clusters 2 and 3 (Figure 2(a)). These clusters contained genes associated with “cortex/forebrain development” and “neuron proliferation” (Figure 2(b)).

Next, we investigated the expression of genes involved in biological processes associated with astrocytes, that is, lactate metabolism, myelination, the BBB, and cholesterol synthesis (Figure 2(c) and Supplemental file Table S1). Genes involved in lactate metabolism were more highly expressed in both forebrain subtypes and in hindbrain GLAST^neg^ astrocytes compared to the other populations (Figure 2(c)). Myelination and cholesterol synthesis genes were highest expressed in spinal cord and forebrain GLAST^pos^ astrocytes (Figure 2(c)). In all populations BBB genes were expressed at similar levels, except for GLAST^pos^ hindbrain astrocytes in which expression was much lower (Figure 2(c)). Cholesterol synthesis genes showed highest expression in all forebrain and spinal cord populations (Figure 2(c)). Of note, hindbrain GLAST^pos^ exhibited higher number of DEGS and lowest expression of all of these gene sets (Figures 1(e) and 2(c)), suggesting they are most distinct from other astrocyte subtypes. Dissecting the differences between hindbrain subtypes further, we found that genes enriched in GLAST^neg^ compared to GLAST^pos^ astrocytes were associated with “myelination” and “oligodendrocyte differentiation” (Figure 2(d) and Supplemental file Table S2). Of note, there were no DEGs detected when directly comparing GLAST^pos^ and GLAST^neg^ forebrain populations (Supplemental file Table S3).

The top 500 expressed genes among all astrocyte populations, representing the core astrocyte transcriptional profile in our data, are listed in supplemental file Table S4. This core astrocyte profile was compared to other published astrocyte profiles (Batiuk et al., 2020; Zeisel et al., 2015; Zhang et al., 2014, 2016), and the overlap with mouse and human astrocyte gene sets was 20–50% (Figure 2(e)). Highest overlap was observed with mouse astrocyte profiles of Zhang et al. and Batiuk et al., and we identified 15 genes overlapping with all investigated mouse astrocyte gene sets (Figure 2(f)). In addition, expression of genes enriched in astrocyte subtypes identified in other studies (Batiuk et al., 2020; Zeisel et al., 2015; Zhang et al., 2014) was analyzed in our astrocyte subtypes (Figure 2(g)). Genes enriched in the mature astrocyte subtype “AST1”, identified by Batiuk et al. (Batiuk et al., 2020) using single‐cell RNA‐seq and associated with subpial and hippocampal regions, were expressed highest in both hindbrain populations. Genes of mature subtypes “AST2‐3”, associated with cortical layers, were highest expressed by both forebrain populations in our dataset (Figure 2(g)). Astrocyte population “AST4” may represent a progenitor population (Batiuk et al., 2020), and genes enriched in this subtype were highest expressed by GLAST^neg^ hindbrain astrocytes (Figure 2(g)). “AST5” is annotated as an intermediate progenitor astrocyte subtype (Batiuk et al., 2020), and was more associated with both forebrain and GLAST^neg^ hindbrain astrocytes (Figure 2(g)). Lin et al. identified five distinct subtypes based on surface protein expression (John Lin et al., 2017). Enriched genes of populations B and C were predominantly expressed by forebrain astrocytes (Figure 2(g)). Population C is strongly associated with synapse organization and is more proliferative than other astrocytes (John Lin et al., 2017). Astrocytes from population C have higher migratory potential than other astrocytes (John Lin et al., 2017), and are more related to both hindbrain and GLAST^neg^ forebrain subtypes (Figure 2(g)). Genes enriched in population D and E were expressed highest in both hindbrain populations (Figure 2(g)). Genes differentially expressed in striatal compared to hippocampal astrocytes (Chai et al., 2017) were predominantly expressed by GLAST^pos^ hindbrain astrocytes (Figure 2(g)). Striatum‐enriched astrocyte genes were also associated with spinal cord astrocytes, whereas hippocampus‐enriched genes were also associated with GLAST^pos^ forebrain astrocytes (Figure 2(g)). These studies focused on astrocytes from the brain, explaining the low correlation of these subpopulations with spinal cord astrocytes (Figure 2(g)).

Summarizing, our findings demonstrate that GLAST^pos^ and GLAST^neg^ astrocytes are transcriptionally distinct and partially overlap with subtypes identified in other studies, indicating that GLAST expression distinguishes distinct astrocyte subtypes.

### 3.3 Transcriptional profiles of astrocyte subtypes differ during EAE

**FIGURE 3:**
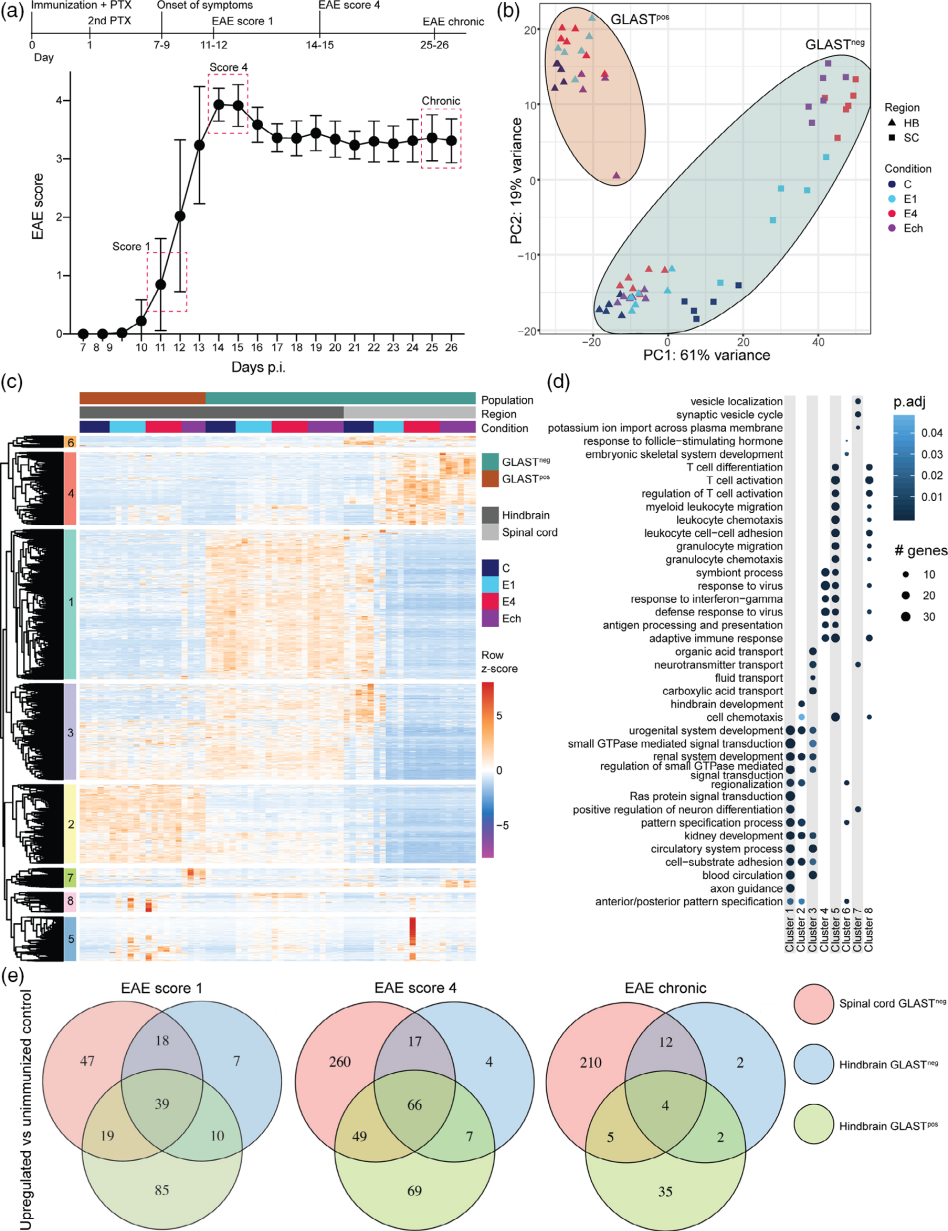
Distinct transcriptional responses of astrocyte subtypes during EAE. (a) Schematic overview of EAE timeline starting with immunization at day 0 (top) and the EAE disease progression from 7–26 days post immunization (p.i.; bottom). Mice were sacrificed at score 1 (E1), score 4 (E4), and chronic (Ech) as indicated with red dotted squares. Unimmunized mice served as control (C). Points indicate mean ± *SD*. (b) Principal component analysis of hindbrain and spinal cord astrocyte subtypes at different EAE stages. (c) Unsupervised clustering of all genes differentially expressed between different conditions within astrocyte populations, illustrated as row z‐scores of normalized counts. (d) GO terms enriched in gene clusters from (c). Top five enriched GO terms per cluster are plotted against enrichment of these GO terms in all clusters. (e) Comparison of upregulated genes among astrocyte subtypes in different EAE stages compared to unimmunized mice

Astrocytes play a major role in EAE development (Brambilla et al., 2014) and transcriptomic changes during EAE are specific to particular regions (Itoh et al., 2017), but subtypes have been poorly explored. To address differences between astrocyte subtypes during EAE, we investigated their gene expression profiles over the course of EAE. Hindbrain and spinal cord astrocytes (GLAST^pos^ and GLAST^neg^) were isolated from unimmunized control animals (C), and during EAE at score 1 (E1: mild clinical signs), score 4 (E4: severe clinical signs), and chronic (Ech: chronic clinical signs) and profiled with RNA‐seq (Figure 3(a)). Since EAE only affects the forebrain only to a minor extent (Constantinescu, Farooqi, O'Brien, & Gran, 2011), we excluded forebrain astrocytes from our analyses.

In line with our previous results, GLAST^pos^ and GLAST^neg^ astrocytes segregated clearly in control animals and during EAE based on PCA (Figure 3(b) and Figure S4). Most variance over the course of disease was observed in spinal cord astrocytes, whereas segregation in both hindbrain subtypes in different EAE stages was less pronounced (Figure S4). Hierarchical clustering of all DEGs between subtypes and different EAE stages revealed three gene clusters (4, 5, and 8) associated with EAE progression (Figure 3(c)). Genes in clusters 5 and 8 were upregulated in all EAE stages and in all subtypes and were associated with “T‐cell activation”, “leukocyte migration”, and other immune‐related processes (Figure 3(d)). Cluster 4 was specifically upregulated in spinal cord astrocytes during EAE and contained genes involved in “response to virus”, “response to interferon‐gamma”, and “antigen‐presentation” (Figure 3(c),(d)). These results show that all astrocyte subtypes acquire an immune‐activated phenotype during EAE.

To further assess differences between astrocyte subtypes during EAE, we investigated the upregulated genes in each subtype per EAE stage, compared to astrocytes from unimmunized control mice (Figure 3(e)). Spinal cord astrocytes had markedly more upregulated genes in every condition compared to both hindbrain populations, and the number of upregulated genes was lowest in hindbrain GLAST^neg^ astrocytes (Figure 3(e)). The overlap of upregulated genes among all populations was markedly low, suggesting distinct transcriptional responses at different stages of EAE (Figure 3(e)).

Our comparisons show that astrocyte subtypes exhibit distinct gene expression profiles over the course of EAE, and that transcriptional changes in spinal cord astrocytes are most pronounced.

### 3.4 Spinal cord astrocytes exhibit a reactive transcriptional profile especially during acute EAE stages

Most EAE‐associated transcriptional changes were detected in spinal cord astrocytes, which is in line with previous observations (Itoh et al., 2017) and EAE pathology, since most lesions occur in the spinal cord (Constantinescu et al., 2011); hence, we focused on this population to further dissect astrocyte changes during EAE in more detail.

**FIGURE 4:**
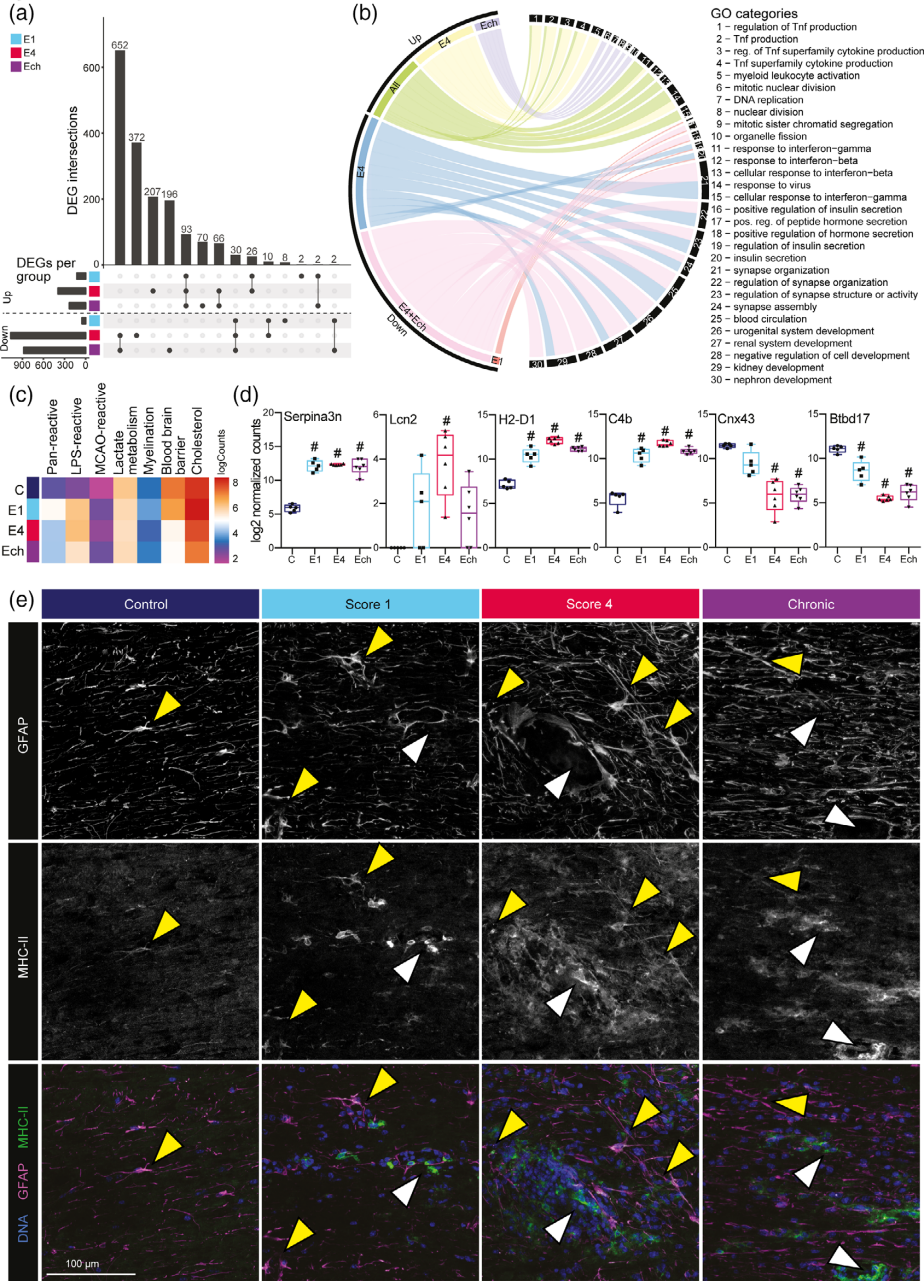
Reactivity of spinal cord astrocytes is most pronounced during acute stage of EAE. (a) Upset diagram depicting the number and overlap of DEGs comparing each EAE stage to unimmunized control. Bars show number of enriched and depleted genes (bottom‐left). Overlapping DEGs are illustrated by interconnected dots between groups (bottom‐right) and the number of DEGs are depicted in in bar graph (top). (b) Circos plot depicting GO annotations of up‐ and down‐regulated genes per EAE stage compared to astrocytes from unimmunized control mice. (c) Mean log expression of pan‐reactive, LPS‐reactive, and MCAO‐reactive astrocyte markers, and genes involved in lactate metabolism, myelination, blood brain barrier, and cholesterol synthesis. For a list of genes see supplemental file Table S1 (*n* = 4–6). (d) Normalized expression of selected reactive astrocyte markers *Serpina3n*, *H2‐D1*, and *Lcn2* and of *C4b*, *Cxn43*, and *Btbd17*. Significantly different expression compared to unimmunized control is indicated (#). Boxes show 25th to 75th percentiles and median, and whiskers indicate min/max (*n* = 4–6). (e) Representative images of MHC‐II (green) and GFAP (magenta) co‐expression in mouse spinal cord during EAE disease progression. Yellow arrows indicate co‐expression; white arrows indicated MHC‐II expression in GFAP^neg^ structures (*n* = 3)

We found most DEGs in E4 and Ech stages compared to unimmunized controls (Figure 4(a)). Interestingly, a considerable number of upregulated (93) and downregulated (30) genes were shared across all EAE stages indicating a partial overlap in transcriptional programs between stages. By clustering all DEGs between EAE stages, we found one main cluster for unimmunized mice, and one main cluster for EAE (Figure S5A). Cluster 1 genes were predominantly expressed in astrocytes from unimmunized mice and were associated with “synapse organization” and “cell chemotaxis” (Figure S5A,B). In all stages of EAE, cluster 2 was highly expressed and genes were enriched for “response to virus” and “response to interferon‐gamma” (Figure S5A,B). Genes that were upregulated in all disease stages and specifically in E4 were associated with immune‐related GO terms such as “Tnf production”, “myeloid leukocyte activation”, and “response to virus” (Figure 4(b)). Genes upregulated in Ech were involved in “mitotic nuclear division” and “DNA replication”, indicating a proliferative astrocyte phenotype in this stage (Figure 4(b)). Downregulated genes in predominantly E4 and Ech were associated with “synapse organization”, “hormone secretion”, and “blood circulation” (Figure 4(b)). The core astrocyte EAE profile with all up‐ and downregulated genes in all disease stages is listed in supplemental file Table S5.

We determined the expression of reported reactive astrocyte genes (Liddelow et al., 2017; Zamanian et al., 2012) and genes involved in known astrocyte functions (Figure 4(c) and Supplemental file Table S1). Pan‐reactive and LPS‐reactive (also known as A1) astrocyte genes were upregulated during all stages of EAE, whereas MCAO‐reactive (also known as A2) astrocyte genes remained lowly expressed (Figure 4(c)). This LPS‐reactive astrocyte signature is associated with neurotoxicity (Liddelow et al., 2017), suggesting that astrocytes during EAE acquire a gene signature in line with a more detrimental phenotype. However, further work is required to delineate the exact function of this reactive astrocyte signature in vivo.

Genes involved in lactate metabolism and myelination did not change dramatically in EAE, whereas the expression of BBB and cholesterol synthesis genes decreased during disease progression (Figure 4(c)). Common astrocyte markers such as *Aldh1l1*, *Slc1a2*, *Cnx43*, *Aqp4*, *Fgfr3* and the previously described (Zhang et al., 2014) but not well‐known astrocyte gene *Btbd17* were downregulated in most EAE stages in spinal cord astrocytes, but not hindbrain astrocytes (Figures 4(d) and S5C). Other markers that increased during EAE were mostly immune‐related and MHC‐II components (*C4b*, *H2‐Aa*, *Cd274*), which were also increased in hindbrain astrocytes, albeit less pronounced (Figure 4(d) and S5C). To verify that MHC‐II is expressed by astrocytes and upregulated during acute EAE, we co‐labelled spinal cord tissue for MHC‐II and GFAP (Figure 4(e)). We observed low MHC‐II expression in astrocytes in unimmunized mice, whereas MHC‐II expression was increased during EAE progression especially at score 4 (Figure 4(e) and S5D).

These data demonstrate that astrocytes acquire a highly reactive transcriptional profile particularly during acute stages of disease, highlighted by upregulation of inflammation and neurotoxic markers, while downregulating genes involved in homeostatic functions.

### 3.5 Astrocytes acquire a more proliferative profile in chronic EAE

**FIGURE 5:**
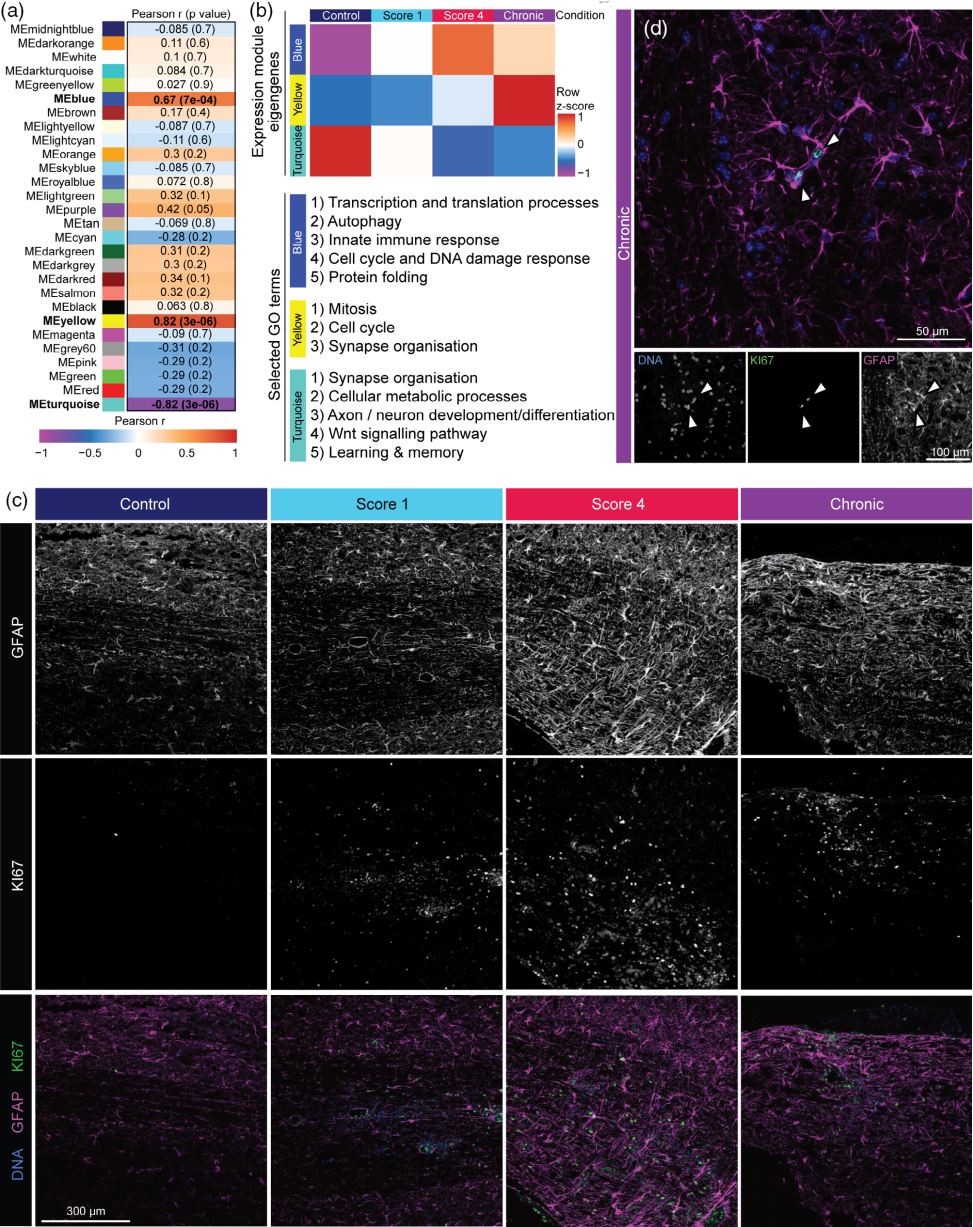
Astrocytes express proliferation markers in chronic EAE. (a) Correlation of module eigengenes with EAE disease progression from unimmunized control to chronic EAE. Numbers indicate Pearson r and p value in brackets. Significant modules are labelled in bold. (b) Mean expression of eigengenes from the blue, yellow, and turquoise modules in different conditions, depicted as row z‐scores (top), and selected enriched GO terms associated with each module (bottom). For a complete list of GO terms per module see supplemental file Table S6. (c, d) Representative images of KI67 (green) and GFAP (magenta) co‐expression in mouse spinal cord during EAE disease progression (c). Enlarged image shows co‐expression in spinal cord of chronic EAE mice (d) (*n* = 3)

To unbiasedly determine gene modules associated with distinct EAE disease stages, we used weighted gene co‐expression network analysis (WGCNA). Based on a consensus network, genes were clustered in 28 modules (Figure 5(a)). Expression of the module eigengenes (ME), or first principal component, of the blue, yellow, and turquoise modules correlated significantly with EAE progression (Figure 5(a)). All GO terms associated with genes in these modules are listed in supplemental file Table S6. MEblue was highest expressed in E4 and moderately in other EAE stages, whereas it was depleted in astrocytes from unimmunized control mice (Figure 5(b)). Genes in this module were involved in “transcriptional and translation processes”, “autophagy”, and “innate immune response” (Figure 5(b)), which is in line with our previous observations. In astrocytes of unimmunized mice, MEturquoise was highest expressed and genes were annotated with “synapse organisation”, “axon/neuron development”, and “learning/memory” (Figure 5(b)). MEyellow was mainly expressed in Ech and genes in this module were associated with “mitosis” and “cell cycle” (Figure 5(b)). Concordantly, genes associated with “DNA replication”, “mitosis”, and “nuclear division” were also present in spinal cord astrocytes in Ech compared to all other stages (Figure 4(b)). To determine if astrocytes are proliferating in chronic EAE, we analyzed co‐expression of KI67 and GFAP in mouse spinal cord. The overall number of KI67 positive cells strongly increased during EAE progression, and also the number of KI67 positive GFAP‐expressing cells increased, especially in chronic EAE (Figure 5(c),(d) and S5D).

In summary, these results suggest that astrocytes acquired a more proliferative profile, which may promote tissue regeneration by glial scar formation.

## 4 DISCUSSION

Here, we demonstrate transcriptional heterogeneity of astrocytes within and across anatomical regions, and that astrocyte subtypes have distinct gene expression profiles during the course of EAE, with most pronounced changes in the spinal cord. Detailed transcriptional characterization of astrocyte subtypes and their differential contribution to disease are largely lacking, and we provide an extensive transcriptional analysis of astrocyte subtypes during EAE progression. Our main findings are that (a) astrocytes in forebrain and hindbrain consist of two transcriptionally distinct subtypes based on GLAST expression (only GLAST^neg^ astrocytes in spinal cord). (b) Astrocytes exhibit substantial regional heterogeneity based on gene expression. (c) Astrocyte subtypes display a differential transcriptional response during EAE, and spinal cord astrocytes show most pronounced changes. (d) Spinal cord astrocytes are highly reactive during acute EAE, downregulate myelination and BBB support genes, and switch to a more proliferative phenotype during chronic EAE.

We identified distinct transcriptional profiles comparing GLAST^pos^ and GLAST^neg^ astrocytes, suggesting they represent distinct astrocyte subtypes. Differences are more pronounced in hindbrain compared to forebrain, and we mainly detected GLAST^neg^ astrocytes in spinal cord. The most significantly enriched gene in hindbrain GLAST^pos^ astrocytes compared to GLAST^neg^ astrocytes is Growth differentiation factor 10 (*Gdf10*). GDF10, a member of the TGF‐β family, is expressed by Bergmann glia, which are unipolar astrocytes in the Purkinje layer of the cerebellum (Koirala & Corfas, 2010). Bergmann glia are essential for neuronal migration during development and are involved in the regulation of synaptic transmission during adulthood. Interestingly, knockout of *Glast* impairs synaptic wrapping by Bergmann glia (Miyazaki et al., 2017), underlining the importance of GLAST for these cells. The population of hindbrain GLAST^pos^ astrocytes appears to be enriched for Bergmann glia, hence our protocol may offer a novel isolation strategy for this astrocyte subtype.

To integrate our findings with previous observations, we compared our identified astrocyte subtypes with published datasets on astrocyte heterogeneity. For example, both forebrain and hindbrain GLAST^neg^ astrocyte transcriptional profiles overlapped with profiles of intermediate progenitor cells (Batiuk et al., 2020); however, this overlap was less pronounced compared to a proliferative astrocyte subtype observed in a different study (John Lin et al., 2017). Integration of findings with multiple studies remains a difficult task due to differences in technology (bulk‐ vs single cell mRNA sequencing), isolation methods, mouse strains, anatomical regions, and availability of data. Yet, detailed comparison with previous findings is essential to advance our understanding and uniform the field of astrocyte heterogeneity.

Our FACS data shows that the frequency of GLAST^pos^ astrocytes decreases from ~40% in forebrain, to ~20% in hindbrain, to less than 1% in spinal cord. Different local CNS environments require distinct support by astrocytes, hence varying frequencies across regions indicate functional differences of astrocyte subtypes. Additionally, this difference in subtype frequencies also demonstrates regional heterogeneity. Regional heterogeneity of astrocytes has been shown previously (Chai et al., 2017; Itoh et al., 2017; John Lin et al., 2017; Lee et al., 1994; Simpson et al., 2011; Yeh, Lee, Gianino, & Gutmann, 2009) and expression follows the dorsoventral axis (Morel et al., 2017). Expanding these observations, our findings suggest that transcription follows the rostrocaudal axis from forebrain to spinal cord. We find *Hox* genes to be expressed in a region‐dependent manner, especially in spinal cord astrocytes, where *Hoxc* genes are enriched. *Hox* genes are involved in embryonic development, where they specify regions in form of segments along the rostrocaudal axis (Pearson, Lemons, & McGinnis, 2005). These genes are also involved in positioning of spinal cord astrocytes (Hochstim, Deneen, Lukaszewicz, Zhou, & Anderson, 2008). Hence, *Hox* genes appear to not only define astrocyte positioning during development, but also shape transcriptional differences across anatomical regions in adulthood.

Delineating astrocyte heterogeneity is of particular importance to understand pathogenic processes, since cell subsets may differentially contribute to disease, as previously established for astrocytes in glioma (John Lin et al., 2017). We found nonoverlapping DEGs and differences in the number of DEGs in GLAST^pos^ and GLAST^neg^ astrocytes in hindbrain, indicating subtype specific transcriptional responses during EAE. Many studies isolate astrocytes using one specific marker (e.g., ACSA, GLAST, GFAP, or ALDH1L1), and since these markers may not be present on the surface of all astrocytes, it is important to consider that a selection for a particular subtype can occur, which will likely skew the results obtained.

One previous study analyzed astrocyte transcriptomes during EAE (Itoh et al., 2017), focusing on one disease stage that is most similar to our chronic stage. Itoh et al. described that most changes occur in spinal cord astrocytes, and that a hallmark of astrocytes during EAE is a reduced expression of cholesterol synthesis genes. Increasing expression of these genes in astrocytes alleviated EAE symptoms (Itoh et al., 2017), indicating a role for astrocyte‐derived cholesterol in EAE severity. We also detected most transcriptional changes in spinal cord astrocytes, which is likely because most lesions occur in this area (Constantinescu et al., 2011). In our study, expression of cholesterol synthesis genes was also decreased, which was most pronounced in the chronic stage.

In the acute stage (E4), we observed a stark increase in neuroinflammatory and LPS‐reactive astrocyte genes, whereas expression of MCAO‐reactive genes was low. These findings are in line with recent observations that a pro‐inflammatory and neurotoxic astrocyte subpopulation expanded during EAE (Wheeler et al., 2020). This reactive astrocyte phenotype is also observed in active MS lesions, reflected by co‐expression of C3 and GFAP, and to a lesser extent also in chronic active and inactive lesions (Liddelow et al., 2017), suggesting this phenotype is mostly present during earlier phases of lesion pathology. Concurrently, astrocytes express MHC‐II around lesions, suggesting they are able to stimulate T‐cell (re)activation. Supporting that hypothesis, other studies demonstrated that astrocytes play a role in the recruitment of peripheral immune cells in EAE (Brambilla et al., 2014; Wang et al., 2013). Astrocytes in active MS lesions contain myelin debris, which they take up through receptor‐mediated endocytosis potentially using lipoprotein receptor‐related protein 1 (LRP1) leading to NFkB activation (Ponath et al., 2017). Thus, astrocytes may present myelin antigens to infiltrating lymphocytes to stimulate (re)activation early during lesion formation.

These reactive astrocytes in early and acute EAE lose their homeostatic signature including cholesterol synthesis, BBB, and neuronal‐support genes. Facilitated entry of immune cells from the blood to the CNS via the BBB is a hallmark of MS (Compston & Coles, 2008; Dendrou, Fugger, & Friese, 2015; Thompson, Baranzini, Geurts, Hemmer, & Ciccarelli, 2018). A downregulation of BBB genes in reactive astrocytes may facilitate transmigration of immune cells from the blood to the CNS. During MS, many astrocytic end feet are lost or retracted and thus do not cover the entire endothelial layer (Brosnan & Raine, 2013; De Parratt & Prineas, 2010), likely rendering the BBB more accessible for cellular transmigration.

Our data furthermore indicate increased proliferation of astrocytes in chronic EAE, which may be relevant for glial scar formation. Glial scar formation, which occurs after demyelination predominantly in chronic MS lesions, can support regeneration of tissue, that is, restoration of BBB function, remyelination, and shielding intact tissue from spreading damages (Ponath, Park, & Pitt, 2018). In line with this argument, astrocytes in remyelinating MS lesions show partly regenerated end feet, although structural abnormalities such as free‐floating astrocytic processes remain (Brosnan & Raine, 2013). Concomitantly, in our dataset reactive astrocyte gene expression decreased in the chronic stage of EAE, which might indicate that astrocytes lost their detrimental signature and acquired more beneficial/regenerative properties.

Overall, astrocytes seemed to lose their homeostatic function in EAE, as was evident from reduced expression of genes involved in lactate metabolism, BBB function, and cholesterol synthesis. We also observed that many common astrocyte markers were decreased in EAE including *Cnx43*, *Btbd17*, *Apoe*, *Aldh1l1*, *Slc1a2*, *Slc1a3*, *Aqp4*, and *Fgfr3*. We propose that in addition to an upregulation of reactive genes, a loss of homeostatic signature genes is a hallmark of reactive astrocytes and should be considered when studying astrocyte reactivity. In summary, we provide evidence that astrocytes are highly reactive and potentially detrimental during acute EAE, whereas they may promote regeneration during recovery.

Interestingly, astrocytes expressed moderate levels of some oligodendrocyte and oligodendrocyte precursor cell (OPC) genes, which were increased during EAE. Expressed genes include *Plp1*, *Mbp*, *Olig1*, and *Olig2*, but not markers such as *Mog*, *Ndrg1*, or *Pdgfra*. This ambiguous expression pattern makes it unlikely that our astrocytes are substantially contaminated by oligodendrocytes/OPCs. In microglia, phagocytosis of myelin can lead to the detection of oligodendrocyte‐derived mRNA molecules (Schirmer et al., 2019), and since reactive astrocytes are able to phagocytose (Morizawa et al., 2017), this could provide an explanation for our findings. To further investigate the presence of oligodendrocyte/OPC transcripts in astrocytes, we employed an available astrocyte gene expression dataset that was obtained through RiboTag technology (Itoh et al., 2017). In this dataset, we found similar expression patterns, where genes thought to be specific for the oligodendrocyte lineage are expressed by astrocytes. These data indicate that it is unlikely that phagocytosis is a significant source of oligodendrocyte/OPC transcripts in astrocytes. Overall, this may indicate that expression of oligodendrocyte/OPC genes in astrocytes is a biological phenomenon. Supporting this notion, a subset of astrocytes derives from OLIG2‐expressing progenitors (Tatsumi et al., 2018), suggesting that oligodendrocytes and astrocytes share a common lineage. Furthermore, astrocytes can transdifferentiate into oligodendrocytes by expression of the transcription factors SOX10 (Khanghahi, Satarian, Deng, Baharvand, & Javan, 2018) or SOX2 (Farhangi, Dehghan, Totonchi, & Javan, 2019), which might be an important mechanism to enhance remyelination after damage. Together, our findings support the emerging concept that astrocytes can obtain oligodendrocyte characteristics, while maintaining a core astrocyte profile.

Our data provides evidence that astrocyte subtypes show a heterogeneous response to EAE, and that particularly spinal cord astrocytes are highly reactive during acute EAE but switch to a more protective role in the chronic stage. In conclusion, we generated a comprehensive transcriptional blueprint of inter‐ and intraregional astrocyte subtypes in homeostatic conditions and during EAE.

## CONFLICT OF INTEREST

The authors declare no competing interests.

## AUTHOR CONTRIBUTIONS

B.J.L.E., C.G., E.W.G.M.B. and S.M.K. contributed to conceptualization. C.G., E.M.W., I.D.V., M.B., N.B. and S.M.K. designed and performed the experiments. M.B. analyzed and visualized the data. B.J.L.E., E.W.G.M.B., J.D.L. and S.M.K. acquired funding. B.J.L.E., E.W.G.M.B. and S.M.K. contributed to supervision. M.B. and S.M.K. wrote the manuscript. All authors revised and edited the manuscript.

## Supporting information


**Appendix** **S1**: Supporting information

**Figure S1** Astrocyte FACS‐isolation gating strategy, spatial expression of GLAST and ACSA, and GO terms enriched in astrocyte subtypes (related to Figures 1 and 2). (A) Representative images of FACS gating strategy. DAPI negative cells were considered viable. The non‐myeloid fraction containing astrocytes was next selected as CD11B^neg^CD45^neg^ events. Further gating and selection of astrocyte subtypes is depicted in Figure 1(b).
(B‐C) Spatial expression of *Glast/ Slc1a3* (top) and *Acsa/ Atp1b2* (bottom) in brain and spinal cord tissue determined by in situ hybridization (data from Allen Brain Atlas [Allen Institute, 2004, 2008; Lein et al., 2007]) (B), and by spatial transcriptomics (data from 10x Genomics for brain (10x Genomics, 2019), and Maniatis et al. for spinal cord (Maniatis et al., 2019)).

**Figure S2** Astrocyte heterogeneity across anatomical regions (related to Figure 1). (A) Volcano plots of the indicated comparisons. Genes labelled with name exhibit log2FoldChange > 6 and *p*_adj_<.001. (n = 3)
(B‐C) Spatial expression of *Hoxa7* (top), *Hoxa9* (middle), and *Hoxc9* (bottom) in brain and spinal cord tissue determined by in situ hybridization (data from Allen Brain Atlas [Allen Institute, 2004, 2008; Lein et al., 2007]) (B), and by spatial transcriptomics (data from 10x Genomics for brain (10x Genomics, 2019), and Maniatis et al. for spinal cord (Maniatis et al., 2019)).
(D) Upset diagram depicting the number and overlap of DEGs. Each region was compared to all other regions. Bars show number of enriched and depleted genes (bottom‐left). Overlapping DEGs are illustrated by interconnected dots between groups and numbers are plotted in bar graph above (right).
(E) Circus diagram depicting GO annotations of enriched and depleted genes per astrocyte subtype compared to all other subtypes.

**Figure S3** in vitro and ex vivo astrocytes exhibit distinct transcriptional profiles (related to Figure 1). (A) Principal component analysis of all ex vivo adult and in vitro neonatal astrocytes. (B) Unsupervised clustering of all genes differentially expressed between ex vivo and in vitro astrocytes, illustrated as row z‐scores of normalized counts. Each column represents one sample, each row one gene. (C) GO terms associated with genes enriched and depleted in in vitro compared ex vivo astrocytes. Numbers behind bars indicate number of genes per GO category.

**Figure S4** Variance of astrocyte subtypes during EAE (related to Figure 3). Principal component analysis of all hindbrain and spinal cord astrocyte subtypes during different stages of EAE.

**Figure S5** Heatmap of spinal cord astrocytes and expression of specific genes in astrocyte subtypes during EAE (related to Figures 4 and 5). (A) Unsupervised clustering of all genes differentially expressed in spinal cord astrocytes between different conditions, illustrated as row z‐scores of normalized counts. (B) GO terms enriched in gene clusters from (A). Top five enriched GO terms per cluster are plotted against enrichment of these GO terms in all clusters. (C) Normalized expression of selected reactive astrocyte markers (*Lcn2*, *Serpina3n*, *H2‐D1*, *Gfap*), inflammatory genes (*H2‐Aa*, *Il1β*, *Cd274*, *Clec7a*), and astrocyte markers (*Apoe*, *Aldh1l1*, *Slc1a2*, *Slc1a3*, *Aqp4*, *Fgfr3*, *Cnx43*, *Btbd17*). Significantly different expression compared to unimmunized control is indicated (#). Boxes show 25th to 75th percentiles and median, and whiskers indicate min/max. (n = 4–6)
(D) Quantification of MHC‐II and KI67 co‐expression with GFAP related to Figures 4(e) and 5(D), respectively. Double positive cells were counted at different stages of EAE. One point represents one mouse. Statistical analysis conducted was a one‐way ANOVA corrected for multiple comparison using Bonferroni. *p < 0.05, **p < 0.01, C = unimmunized control, E1 = EAE score 1, E4 = EAE score 5, Ech = Chronic EAE

**Table S1** Lists of astrocyte function genes and reactive astrocyte genes.
**Table S2**. Differentially expressed genes between hindbrain GLAST^neg^ and hindbrain GLAST^pos^.
**Table S3**. Number of differentially expressed genes between astrocyte subtypes.
**Table S4**. Core astrocyte profile. Top 500 expressed genes among all astrocyte subtypes.
**Table S5**. Astrocyte EAE core signature. Genes that are up‐ or down‐regulated in spinal cord astrocytes in all stages of EAE compared to unimmunized mice.
**Table S6**. Gene ontology analysis of genes in significantly correlating WGCNA modules blue, yellow, and turquoise.

## Data Availability

Data are available at NCBI GEO under accession number GSE136358.
