# Supplementary figures and images for: Regionally diverse astrocyte subtypes and their heterogeneous response to EAE
Source: Glia. 2020 Dec 17;69(5):1140–54. doi: 10.1002/glia.23954 (PMC7985878; doi:10.1002/glia.23954)

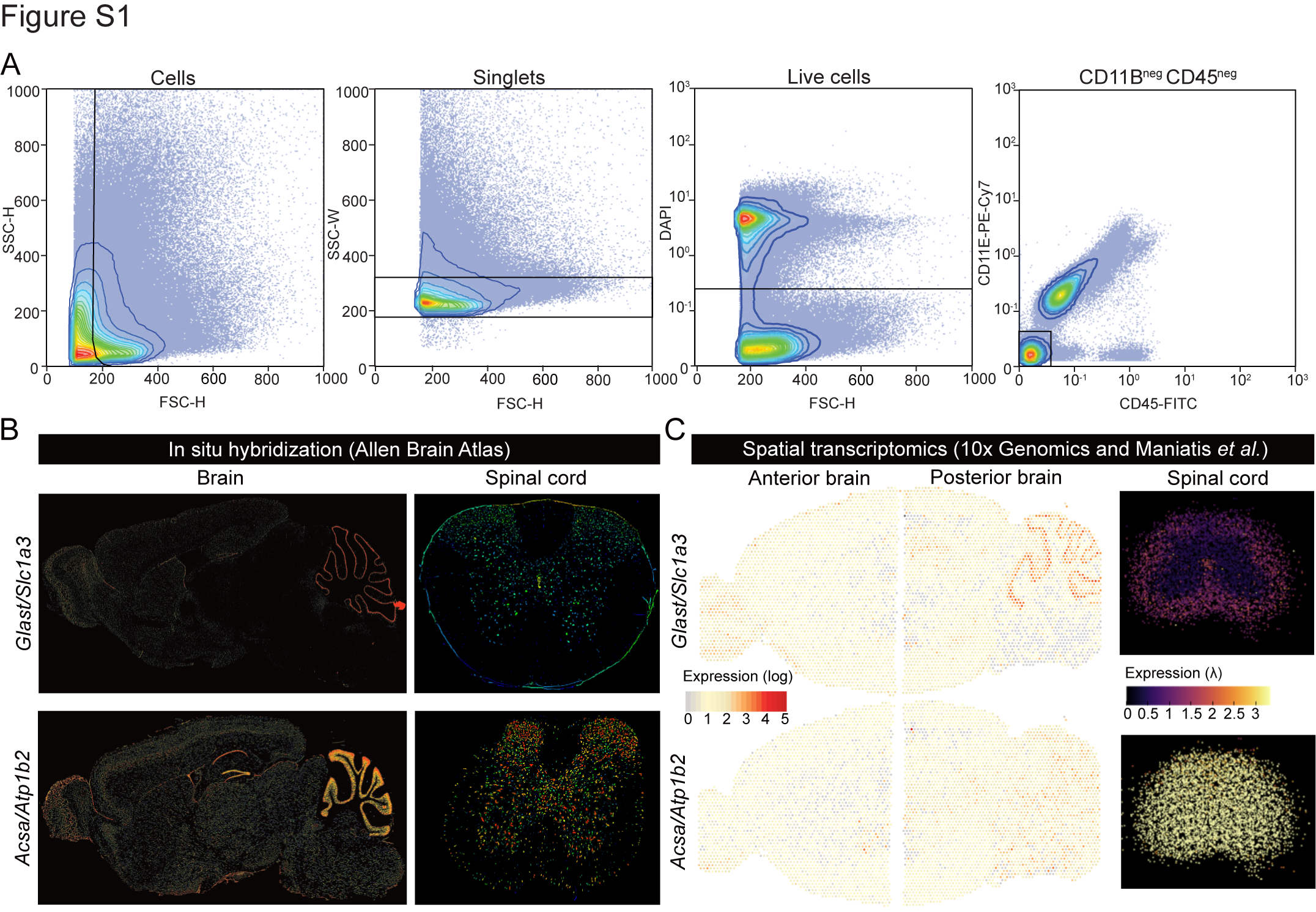

Supplement: Supplementary file 2 — Figure S1 Astrocyte FACS‐isolation gating strategy, spatial expression of GLAST and ACSA, and GO terms enriched in astrocyte subtypes (related to Figures 1 and 2). (A) Representative images of FACS gating strategy. DAPI negative cells were considered viable. The non‐myeloid fraction containing astrocytes was next selected as CD11BnegCD45neg events. Further gating and selection of astrocyte subtypes is depicted in Figure 1(b). (B‐C) Spatial expression of Glast/ Slc1a3 (top) and Acsa/ Atp1b2 (bottom) in brain and spinal cord tissue determined by in situ hybridization (data from Allen Brain Atlas [Allen Institute, 2004, 2008; Lein et al., 2007]) (B), and by spatial transcriptomics (data from 10x Genomics for brain (10x Genomics, 2019), and Maniatis et al. for spinal cord (Maniatis et al., 2019)). [file GLIA-69-1140-s001.tif]

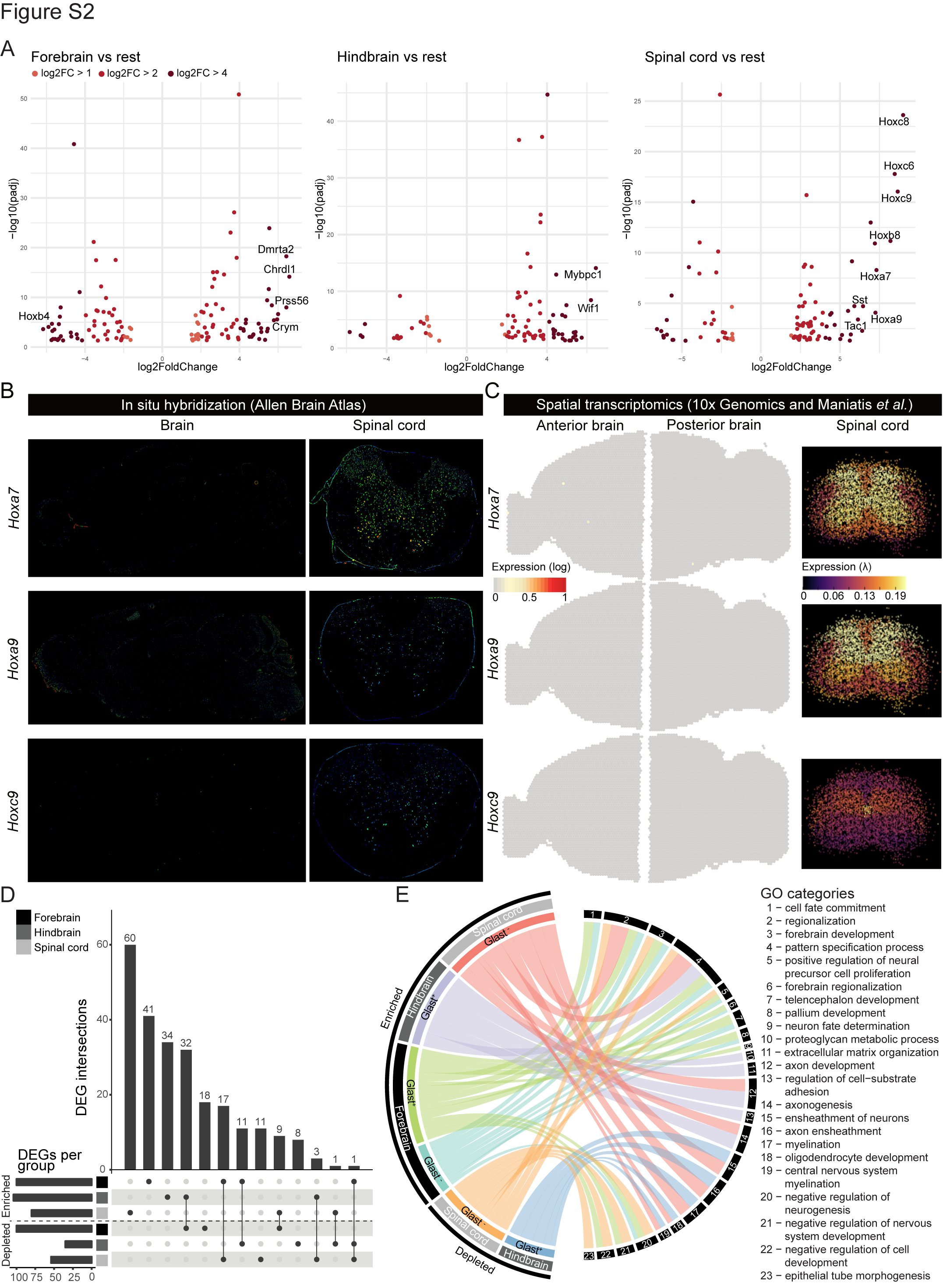

Supplement: Supplementary file 3 — Figure S2 Astrocyte heterogeneity across anatomical regions (related to Figure 1). (A) Volcano plots of the indicated comparisons. Genes labelled with name exhibit log2FoldChange > 6 and p adj<.001. (n = 3) (B‐C) Spatial expression of Hoxa7 (top), Hoxa9 (middle), and Hoxc9 (bottom) in brain and spinal cord tissue determined by in situ hybridization (data from Allen Brain Atlas [Allen Institute, 2004, 2008; Lein et al., 2007]) (B), and by spatial transcriptomics (data from 10x Genomics for brain (10x Genomics, 2019), and Maniatis et al. for spinal cord (Maniatis et al., 2019)). (D) Upset diagram depicting the number and overlap of DEGs. Each region was compared to all other regions. Bars show number of enriched and depleted genes (bottom‐left). Overlapping DEGs are illustrated by interconnected dots between groups and numbers are plotted in bar graph above (right). (E) Circus diagram depicting GO annotations of enriched and depleted genes per astrocyte subtype compared to all other subtypes. [file GLIA-69-1140-s003.tif]

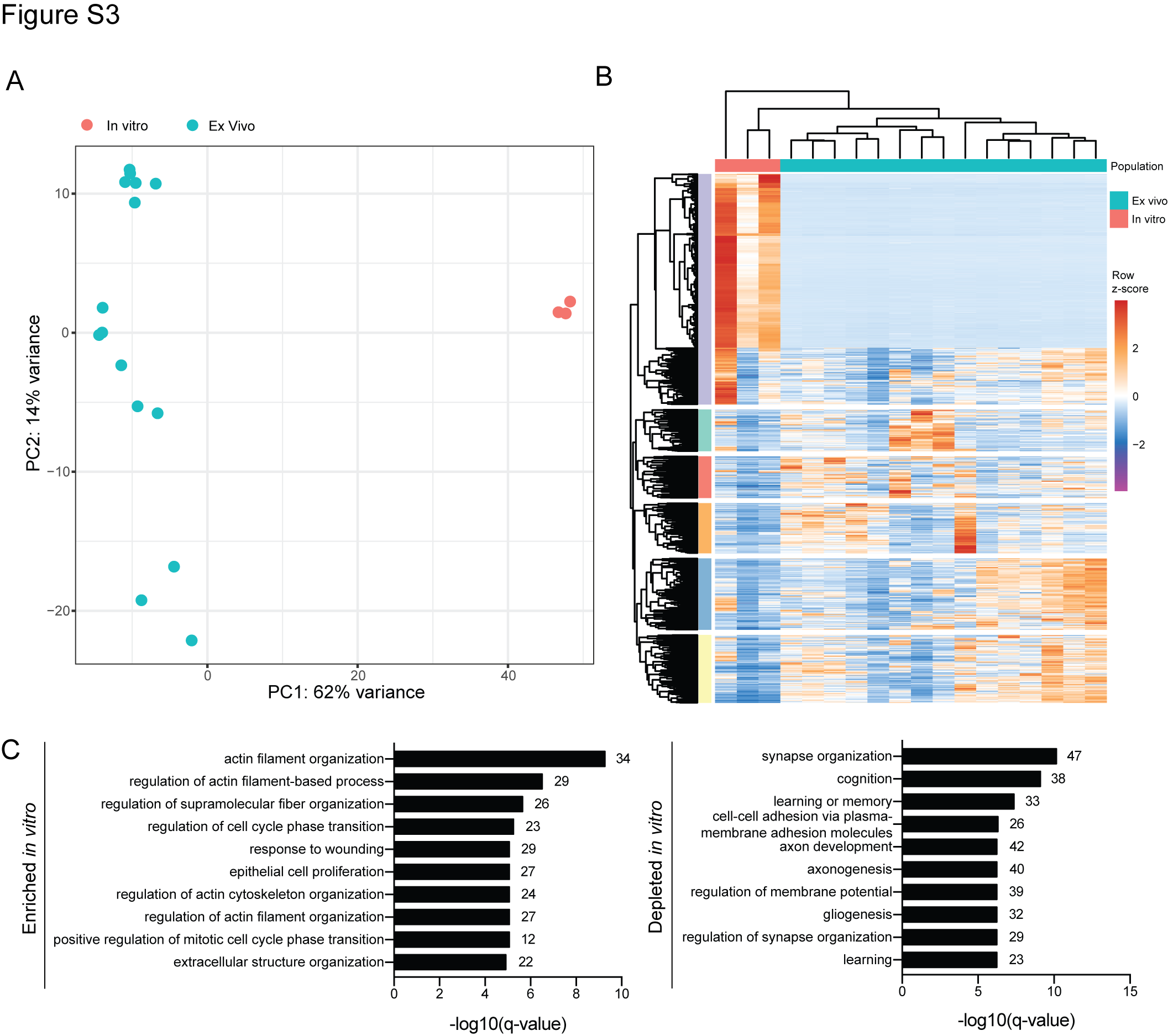

Supplement: Supplementary file 4 — Figure S3 in vitro and ex vivo astrocytes exhibit distinct transcriptional profiles (related to Figure 1). (A) Principal component analysis of all ex vivo adult and in vitro neonatal astrocytes. (B) Unsupervised clustering of all genes differentially expressed between ex vivo and in vitro astrocytes, illustrated as row z‐scores of normalized counts. Each column represents one sample, each row one gene. (C) GO terms associated with genes enriched and depleted in in vitro compared ex vivo astrocytes. Numbers behind bars indicate number of genes per GO category. [file GLIA-69-1140-s004.tif]

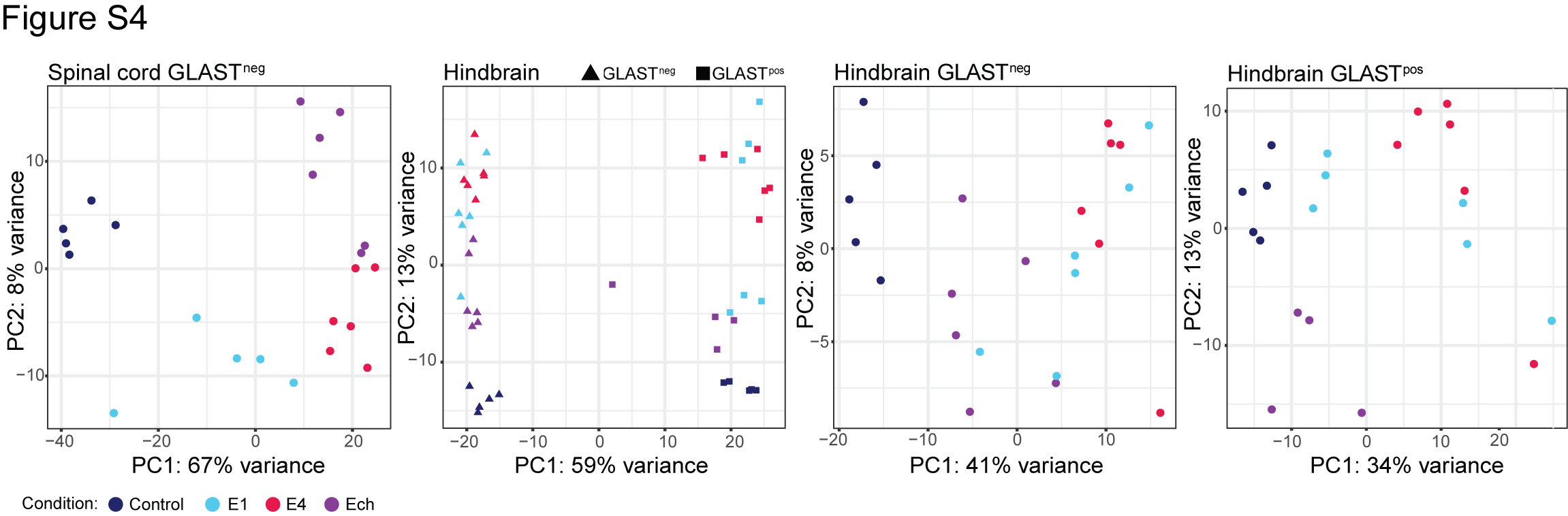

Supplement: Supplementary file 5 — Figure S4 Variance of astrocyte subtypes during EAE (related to Figure 3). Principal component analysis of all hindbrain and spinal cord astrocyte subtypes during different stages of EAE. [file GLIA-69-1140-s005.tif]

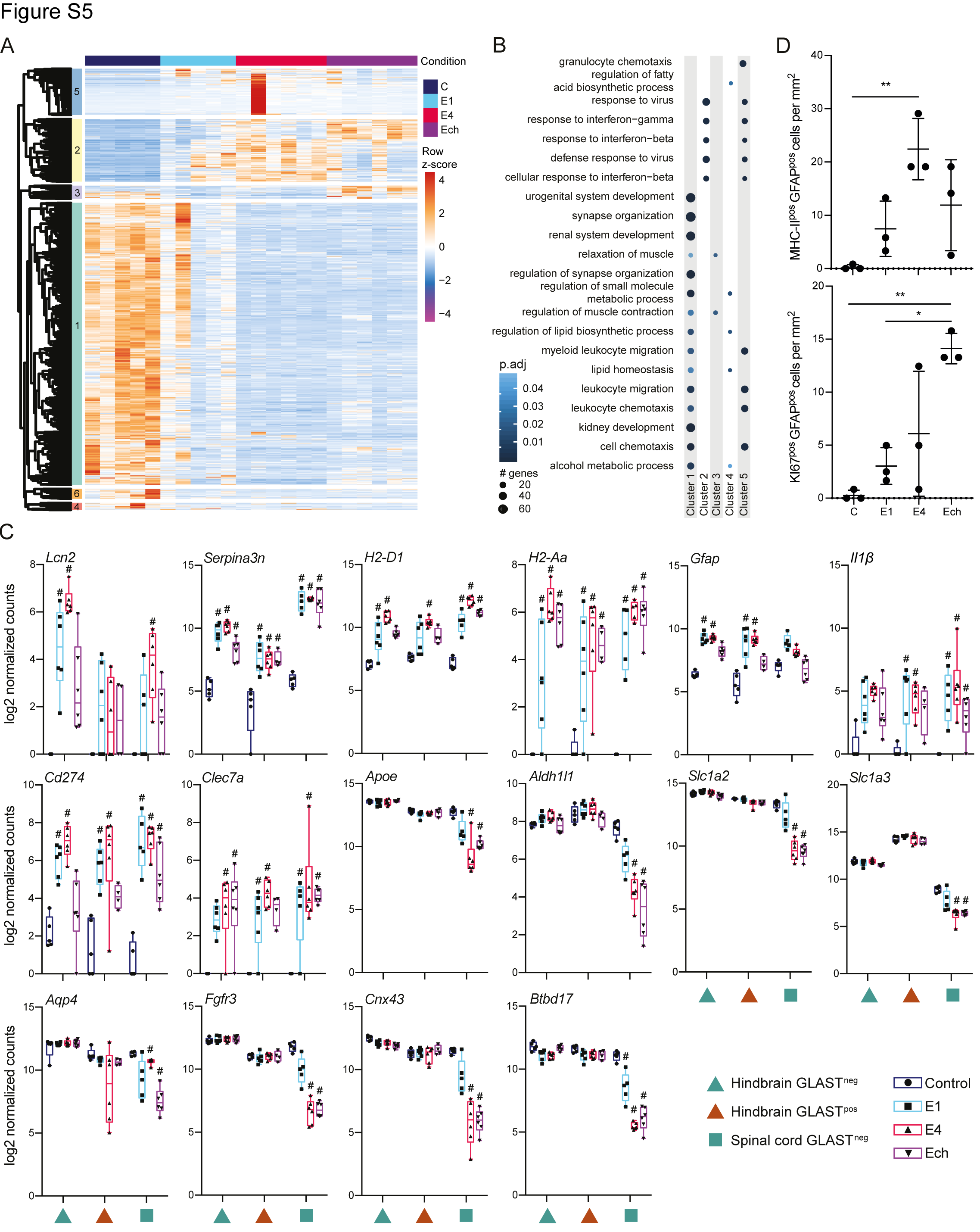

Supplement: Supplementary file 6 — Figure S5 Heatmap of spinal cord astrocytes and expression of specific genes in astrocyte subtypes during EAE (related to Figures 4 and 5). (A) Unsupervised clustering of all genes differentially expressed in spinal cord astrocytes between different conditions, illustrated as row z‐scores of normalized counts. (B) GO terms enriched in gene clusters from (A). Top five enriched GO terms per cluster are plotted against enrichment of these GO terms in all clusters. (C) Normalized expression of selected reactive astrocyte markers (Lcn2, Serpina3n, H2‐D1, Gfap), inflammatory genes (H2‐Aa, Il1β, Cd274, Clec7a), and astrocyte markers (Apoe, Aldh1l1, Slc1a2, Slc1a3, Aqp4, Fgfr3, Cnx43, Btbd17). Significantly different expression compared to unimmunized control is indicated (#). Boxes show 25th to 75th percentiles and median, and whiskers indicate min/max. (n = 4–6) (D) Quantification of MHC‐II and KI67 co‐expression with GFAP related to Figures 4(e) and 5(D), respectively. Double positive cells were counted at different stages of EAE. One point represents one mouse. Statistical analysis conducted was a one‐way ANOVA corrected for multiple comparison using Bonferroni. *p < 0.05, **p < 0.01, C = unimmunized control, E1 = EAE score 1, E4 = EAE score 5, Ech = Chronic EAE [file GLIA-69-1140-s002.tif]
