# Supplementary material for: Regionally diverse astrocyte subtypes and their heterogeneous response to EAE
Source: Glia. 2020 Dec 17;69(5):1140–54. doi: 10.1002/glia.23954 (PMC7985878; doi:10.1002/glia.23954)
Supplement: Supplementary file 1 — Appendix S1: Supporting information [file GLIA-69-1140-s007.docx]

**Below a point by point response to the reviewer’s comments:**

**Reviewer: 1**

*In Borggrewe et al’s study titled Regionally diverse astrocyte subtypes and their heterogeneous response to EAE, the authors elegantly describe GLAST+ and GLAST- astrocyte populations in the forebrain, hindbrain, and spinal cord of mice studied with RNA sequencing. After identifying gene and pathway differences between these subtypes, they focus on spinal cord GLAST- astrocytes and demonstrate how these cells respond to various stages of EAE, with the logical yet interesting finding that astrocytes in chronic stages of the disease express proliferative genes, which may contribute to the glial scar formation. 

Through the manuscript, the authors are often thorough and convincing in their objectives, and they provide solid evidence that supports their claims. Additionally, the novel techniques and analytical tools used throughout the manuscript effectively portray the authors findings that astrocyte subpopulations exist and differ over the EAE disease course. Despite the impressive work, a few questions and comments remain. Primarily, the true identity of the cells is not absolutely convincing, and the lack of association to the human disease (multiple sclerosis) leaves the reader with some questions about the impacts of the data.*

We thank reviewer 1 for the thorough evaluation of our manuscript. As indicated in the point-by-point response, we have added new data and analyses to verify the astrocyte identity of our isolated cells and added a section to the discussion to evaluate the implication of our findings for multiple sclerosis.

*Comments on specific figures:
Figure 1: Figure 1C demonstrates that the selection criteria for astrocytes selects against populations of neurons, oligodendrocytes, and microglia; however, according to the publication referenced in the text (Kantzer et al, 2017), however, this method does not restrict against a number of cells, including radial glia, neural stem cells, and perhaps ependymal cells. The validity of the paper would benefit from a more thorough demonstration that the present cells are astrocytes, both at the gene level, but also histologically. The authors explain that they cultured these cells, so inclusion of staining (GFAP, S100B, or other makers) would enhance this aspect of the paper. Additionally, the authors could cross-check their data by using a fluorescent transgene system. Although not all of these methods are necessary, one additional level of validation should be employed.*

We have performed a new analysis to include genes specific for radial glia cells, neural stem cells, and ependymal cells and replaced Figure 1C with this new graph. Expression of markers for astrocytes is high, whereas expression of markers for other CNS cell types is low (Figure 1C).

To clarify, the cultured astrocytes are from a different source, they are derived from neonatal mouse brains and are not isolated from adult mice using our FACS protocol. Therefore, staining of these cultured astrocytes will provide limited information about the validity of our FACS isolation approach.

It is a good suggestion to cross-check the validity of our approach using a transgene system. This has been done previously by Batiuk et al. in 2017 (Figure A below). These data show that virtually all Aldh1l1-GFP astrocytes expressed ACSA-2.

**Figure A. Figure from Batiuk et al. 2017.**

*- Figure 1E: The authors describe the spinal cord astrocytes as the most transcriptionally distinct population. The Upset Diagram shows that Glast+ HB astrocytes have the most DEGs up- and down-regulated. The authors should better clarify how this observation does not necessarily indicate that the HB is more distinct than the spinal cord, because there is currently no explanation of the data.*

We have done 2 distinct comparisons: astrocytes from different anatomical regions (Figure S2D) and astrocyte subtypes based on GLAST expression and anatomical regions (Figure 1E). In the text, we refer to differences between anatomical regions and state that astrocytes from spinal cord are most distinct compared to hindbrain and forebrain, which is reflected in Figure S2D. When comparing all individual subtypes, the GLAST+ hindbrain astrocytes indeed are most distinct based on number of DEGs. We have adjusted the text (lines 290-291) to resolve this ambiguity.

*Figure 2:
- Figure 2B: Where is Cluster 6?*

Cluster 6 is not shown since it does not exhibit any significant gene ontology terms. We have added to the figure legend of Figure 2B that “Only clusters with significantly enriched GO terms are shown.”

*- Figure 2C: The uniqueness of the Glast+ HB cells seems to be underplayed. They have the most DEGs compared to other samples, and here they have low expression of genes associated with all critical astrocyte functions. The reader would benefit from comments on these findings.*

We agree that it is important to point out the uniqueness of GLAST+ hindbrain astrocytes. Therefore, we added a sentence to the results pointing out the difference of this subtype to the other subtypes (lines 337-339).

*o Figure 2C: The authors should re-define the circles/squares/triangles as well.*

We appreciate this suggestion and have adjusted the figure accordingly.

*- Figure 2G: This comparison is an effective way for the authors to compare their data to other publications. They explain that HB Glast- express AST4 (progenitor markers), but they also express mature AST1 and intermediate AST5. The authors focus on the AST4, but it seems to lead the reader one way while the data is not definitive. Similarly, HB Glast- have low expression of Lin Group C, which is proliferative. If HB Glast- were indeed progenitors, they may be expected to express proliferative markers. These possibilities are interesting, yet the authors do not comment on the controversial/interesting findings. Adding some of these points to the discussion would add to the authors’ opinions that the distinction between cell populations is important.*

We agree with the reviewer that this comparison should be pointed out and discussed more. We believe that integrating these findings with other studies is important to uniform the field. Therefore, we added a part to the discussion evaluation our findings in comparison with these other studies (lines 520-529).

*Figure 3:
Figure 3B: The authors should define was “C” is in the text.*

We added a definition of C (unimmunized control animals) to the results text (line 387) and added an explanation of this abbreviation to the Figure legend (Figure 3).

*Fig 4: This figure begins to focus only on spinal cord astrocytes. Can the authors perform MHCII staining of non-spinal cord astrocytes? Even if data is non-significant, this would still provide insight into the specific pathogenesis of EAE. Additionally, could they demonstrate if this specificity is GLAST+ or GLAST- specific? This would expand the impact of the paper by relating it more to multiple sclerosis (rather than just spinal cord investigation), which I will explain for the discussion section.*

We focused on spinal cord from this figure, as the spinal cord is the region of the CNS that is primarily affected by EAE in mice. To investigate MHCII expression by astrocytes in the brain, as suggested by the reviewer, we performed MHCII staining on brains of EAE mice (Fig B). We included control animals and mice with EAE score 4, the stage where we detected highest expression of MHCII in the spinal cord and in both cases, we did not detect MHCII positive astrocytes.

**Figure B. MHC-II expression by astrocytes in mouse brain during EAE.** Representative images of MHC-II (green) and GFAP (magenta) co-expression in mouse brain regions (medulla, cerebellum, midbrain, hippothalamus, hippocampus, and cortex) during EAE score 4 and unimmunized control mice. (n=3)

*- Fig 4E: Please give quantitative averages of multiple fields with statistical significance, rather than (or in addition to) one representative image.

Fig 5: Again, can the authors give quantitative average, rather than (or in addition to) representative images.*

We quantified the number of MHC-II and KI67 positive astrocytes and added this analysis to Figure S5D.

*Discussion: The discussion would benefit from discussion how these findings could implicate multiple sclerosis treatment or understanding. The current data only studies EAE samples, and the majority of the data investigates spinal cord astrocytes. The initial segregation of cells between Glast+ and Glast- and anatomic segregation is lost by the end of the paper (except for some supplemental figures). If the authors want to make their findings more impactful and more interesting to a broader range of researchers, they should discuss the implications that this has on multiple sclerosis.*

We appreciate this suggestion by the reviewer and changed the discussion accordingly. We added multiple parts discussing the implications of our findings on multiple sclerosis (lines 569-603).


**Reviewer: 2**

*Comments to the Author
This paper explores and expands upon the emerging field of astrocyte diversity under normal and injury conditions. Building off of recent studies that identified molecularly distinct subsets of astrocytes, this study provides additional and important evidence that astrocytes in the adult brain exhibit molecular heterogeneity. Using FACS-based approaches, that do not rely on transgenic reporter,, the authors molecularly define subset of astrocytes in the forebrain, hindbrain, and spinal cord based on ACSA2- and Glast- antibodies. They also assess these populations after EAE and find that that these astrocyte-subsets exhibit marked differences in the spinal cord. In general, the demonstration that these subsets of astrocytes alter their molecular properties after EAE challenge is interesting and worth documenting. 

This is an interesting paper, that will add additional evidence that astrocyte populations in the brain exhibit heterogeneity. The authors do an excellent job of comparing and contrasting their data with existing data on this topic. While this paper is largely descriptive, the profiling studies after EAE will serve as an excellent resource for the field. Overall, this paper suitable for publication in Glia as it adds to emerging evidence that astrocytes exhibit molecular heterogeneity and that this heterogeneity exists (and appears to change) during inflammatory disease. 

I have one comment that I hope the authors will take under advisement prior to publication. In lines 423-430 and figure 4C they mention A1/A2 polarized reactive astrocyte subtypes. I would strongly recommend removing these data, as this nomenclature is no longer valid and any mention of these “populations” is dubious.*

We thank the reviewer for the assessment of our manuscript and appreciate the suggestion. We agree that the A1/A2 nomenclature is obsolete, however, we strongly believe that the comparison of EAE astrocytes with other reactive astrocytes profiles is informative. Therefore, we kept the data, but removed the A1/A2 nomenclature. Instead, we use the actual stimulus that generated the gene expression profiles of “A1/A2” astrocytes as nomenclature, namely LPS and MCAO, respectively (Figure 4C and lines 440-443; 566-567).
